# Tumor-driven stabilization of CD8^+^ T cell exhaustion and implications for cancer immunotherapy

**DOI:** 10.3389/fimmu.2026.1862431

**Published:** 2026-06-26

**Authors:** Romane Thouenon, Marco Ongaro, Grégory Verdeil

**Affiliations:** 1 Department of Fundamental Oncology, University of Lausanne, Lausanne, Switzerland; 2 Ludwig Institute for Cancer Research, University of Lausanne, Lausanne, Switzerland

**Keywords:** T cell exhaustion, cancer, tumor microenvironment, immunotherapies, transcriptional imprinting

## Abstract

CD8^+^ T cell exhaustion is a conserved differentiation program induced by persistent antigen stimulation and originally characterized in chronic infection. In cancer, this program is actively reinforced and stabilized by the tumor microenvironment. Here, we examine how tumors convert a physiological adaptation to chronic stimulation into a deeply entrenched dysfunctional state. Sustained TCR signaling initiates exhaustion through NFAT- and TOX-dependent transcriptional remodeling, but tumor-specific extrinsic pressures, including hypoxia, metabolic competition, ionic imbalance, mechanical stress, and heterogeneous antigen exposure, amplify and stabilize this program. These environmental cues converge on transcription factor networks such as IRF, BATF, NR4A, and NFAT5, which integrate chronic signaling with stress-adaptive responses and progressively restrict effector potential. Exhaustion in tumors becomes epigenetically imprinted. Exhaustion-specific enhancer landscapes persist despite PD-1 blockade, reflecting a lineage-like state enforced by chromatin remodeling factors such as TOX. Thus, checkpoint inhibition transiently restores function without fully reprogramming cellular identity. We propose that tumor-induced exhaustion arises from the layered convergence of chronic antigen signaling and microenvironmental reinforcement, culminating in chromatin fixation. Understanding this stabilization process reframes therapeutic strategies: effective cancer immunotherapy will likely require combinatorial approaches that target not only inhibitory receptors but also metabolic resilience, stress-sensing pathways, and epigenetic architecture. By dissecting how tumors convert adaptive restraint into durable dysfunction, new avenues may emerge to destabilize exhaustion and restore durable antitumor immunity.

## 1 Introduction

CD8^+^ T lymphocytes are essential mediators of adaptive immunity, responsible for recognizing and eliminating infected or transformed cells through antigen-specific cytotoxic responses. Upon acute antigen encounter in an inflammatory context, naïve CD8^+^ T cells undergo clonal expansion and differentiate into effector cells capable of producing interferon-γ (IFN-γ), tumor necrosis factor (TNF), and interleukin-2 (IL-2), while acquiring cytolytic molecules such as perforin and granzymes. Following antigen clearance, most effector cells undergo contraction, leaving a durable memory pool poised for rapid recall responses. This classical effector/memory differentiation program, however, is fundamentally altered when antigen exposure is persistent (1, 2). Under conditions of chronic stimulation, most prominently during chronic viral infections and cancer, CD8^+^ T cells progressively enter a distinct differentiation state termed T cell exhaustion. Originally described in chronic lymphocytic choriomeningitis virus (LCMV) infection models (3), exhaustion is now recognized as a hallmark of tumor-infiltrating lymphocytes (TILs) and a major obstacle to effective antitumor immunity (1, 4). Rather than representing simple dysfunction or deletion, exhaustion constitutes a regulated and structured differentiation program that emerges in response to sustained antigenic signaling (1, 5).

Exhaustion develops progressively and is characterized by a hierarchical loss of effector functions. Early stages involve diminished IL-2 production and reduced proliferative capacity, followed by impaired TNF secretion and, at more advanced stages, compromised IFN-γ production and cytotoxicity (3, 6). Concomitantly, exhausted T cells display sustained expression of multiple inhibitory receptors (IRs), including PD-1, CTLA-4, LAG-3, TIM-3, and others (3, 7). Although inhibitory receptors are transiently upregulated during normal T cell activation, their persistent and combinatorial expression under chronic stimulation is a defining feature of exhaustion (7). Importantly, exhausted T cells retain partial functionality and are not inert. They may continue to exert limited cytotoxic activity and contribute to pathogen or tumor control, albeit insufficiently (3, 8). This residual function distinguishes exhaustion from anergy or deletion and underscores its nature as an adaptive differentiation program rather than a terminally inactive state (1, 5). At the molecular level, exhaustion is defined by a distinct transcriptional and epigenetic landscape. Genome-wide analyses have demonstrated that exhausted T cells possess coordinated alterations in gene modules governing T cell receptor (TCR) signaling, metabolism, survival, migration, and effector differentiation (9, 10). These transcriptional changes are reinforced by stable epigenetic remodeling, including exhaustion-specific chromatin accessibility patterns (11, 12). Notably, many of these epigenetic features persist even after immune checkpoint blockade, indicating that exhaustion represents a relatively stable lineage commitment rather than a transient unfunctional condition (11).

Sustained TCR signaling is the primary driver of exhaustion and initiates a complex transcriptional network (1, 5). Central to this process are members of the nuclear factor of activated T cells (NFAT) family. In acute responses, NFAT1/2 cooperates with AP-1 transcription factors (Fos/Jun) to induce effector-associated genes. Under chronic stimulation, however, NFAT1/2 activity becomes uncoupled from AP-1, promoting the expression of inhibitory receptors and exhaustion-associated genes (13, 14). Experimental models in which NFAT1/2 is constitutively active but unable to interact with AP-1 recapitulate key aspects of the exhaustion transcriptional program, illustrating how altered transcription factor partnerships influence cell fate (13). A major advance in the field was the identification of thymocyte selection-associated high-mobility group box (TOX) as a central regulator of exhausted T cell differentiation (14–18). Acting downstream of sustained NFAT1/2 signaling, TOX orchestrates widespread epigenetic remodeling and stabilizes exhaustion-associated gene expression, including increased accessibility at the *Pdcd1* locus. While deletion of TOX reduces inhibitory receptor expression and partially restores effector function, TOX-deficient T cells fail to persist under chronic antigen exposure, highlighting the dual role of exhaustion programs in limiting overstimulation while preserving cell survival (5–17). Additional transcription factors contribute to shaping the exhausted phenotype. The balance between EOMES and T-bet influences differentiation outcomes, with a high EOMES/T-bet ratio associated with terminal exhaustion (19). TCF-1, encoded by *Tcf7*, is critical for maintaining progenitor-like exhausted populations and restraining terminal differentiation (20, 21). NR4A family members reinforce inhibitory receptor expression and effector attenuation (22). Together, these factors establish a coordinated regulatory network that defines the exhausted lineage.

Exhausted CD8^+^ T cells are not a homogeneous population but comprise distinct subsets organized along a differentiation continuum. A progenitor exhausted subset (T_PEX_) has been identified in both chronic infection and cancer (20, 21, 23). These cells are characterized by intermediate PD-1 expression, TCF-1 expression, absence of TIM-3, and memory-like properties. T_PEX_ retain proliferative potential and self-renewal capacity, functioning as a reservoir that sustains downstream exhausted populations (20, 23). In contrast, terminally exhausted T cells (T_EX_) exhibit high expression of multiple inhibitory receptors, loss of TCF-1, increased EOMES, reduced T-bet, diminished proliferative capacity, and limited longevity (21, 23). Although functionally constrained, T_EX_ may still produce low levels of cytotoxic molecules upon stimulation. Developmental models propose that persistent antigen drives the differentiation of T_PEX_ into progressively more terminal states, potentially through intermediate subsets (23, 24). Despite differences among proposed models, a consistent principle emerges: exhaustion is hierarchically organized, with a stem-like compartment sustaining more differentiated populations. Importantly, immune checkpoint blockade preferentially expands the progenitor compartment, providing a mechanistic explanation for the dependence of therapeutic efficacy on T_PEX_ abundance (21, 25). Clinical studies in melanoma have demonstrated that the presence of TCF-1^+^ progenitor-like exhausted T cells correlates with improved responses to PD-1 blockade (26), underscoring the therapeutic relevance of exhaustion heterogeneity. From an evolutionary perspective, exhaustion likely represents a protective adaptation to chronic antigen exposure. During persistent viral infection, uncontrolled effector activity can result in severe immunopathology. By attenuating effector functions while maintaining partial control over antigen persistence, exhaustion establishes a balance between immune-mediated damage and pathogen containment (1, 5, 27). Experimental disruption of exhaustion pathways can enhance effector responses but may compromise T cell persistence or increase tissue damage, emphasizing that exhaustion is not merely detrimental but context-dependent (15, 27).

Exhausted T cells arising in chronic viral infection and in cancer share a conserved molecular core. Persistent antigen stimulation, sustained inhibitory receptor expression, TOX-dependent epigenetic remodeling, NFAT1/2-driven transcriptional reprogramming, and hierarchical organization into progenitor and terminal subsets are observed in both settings (1, 15, 20). Transcriptomic analyses reveal substantial overlap in exhaustion-associated gene signatures across infection and tumor models, supporting the concept of a common differentiation framework induced by chronic TCR engagement (9, 26). However, important contextual distinctions exist. Chronic viral infections typically involve defined pathogen-derived antigens, systemic inflammation, and relatively predictable antigen kinetics (3, 5). In contrast, cancer presents a heterogeneous and evolving antigenic landscape composed of neoantigens, self-antigens, and differentiation antigens, often accompanied by variable co-stimulatory and inflammatory signals (4, 26). Antigen presentation in tumors may be spatially restricted and dynamically regulated, contributing to heterogeneous T cell stimulation. Moreover, whereas chronic infection occurs within physiological tissue architecture, tumors establish aberrant microenvironments characterized by altered stromal organization, metabolic stress, and immunoregulatory networks (4, 28). Thus, while exhaustion in cancer and chronic infection shares foundational molecular mechanisms, the tumor context imposes additional regulatory constraints that shape T cell fate in distinct ways. Conceptually, exhaustion in chronic infection can be viewed as an adaptive response to persistent pathogen-derived antigen in an inflammatory environment, whereas exhaustion in cancer arises in response to chronic self- or neoantigen exposure within a dysregulated tissue niche. These qualitative differences likely influence progenitor maintenance, terminal differentiation, and responsiveness to immunotherapy (29–31).

Given the conserved core features of exhaustion and the contextual differences imposed by the tumor milieu, it is critical to distinguish universal mechanisms from tumor-specific regulatory inputs. In this review, we examine how the canonical exhaustion program, defined in chronic infection models, is induced and maintained within the cancer setting, and how tumor-intrinsic and microenvironmental factors uniquely modulate this process. By dissecting the intersection between conserved differentiation pathways and tumor-specific regulatory cues, we aim to provide a framework for understanding how exhaustion is shaped in cancer and how it may be therapeutically reprogrammed to achieve durable antitumor immunity.

## 2 Tumor microenvironment specific T cell exhaustion inducers and maintainers

### 2.1 Hypoxia and HIF signaling

**Figure 1:**
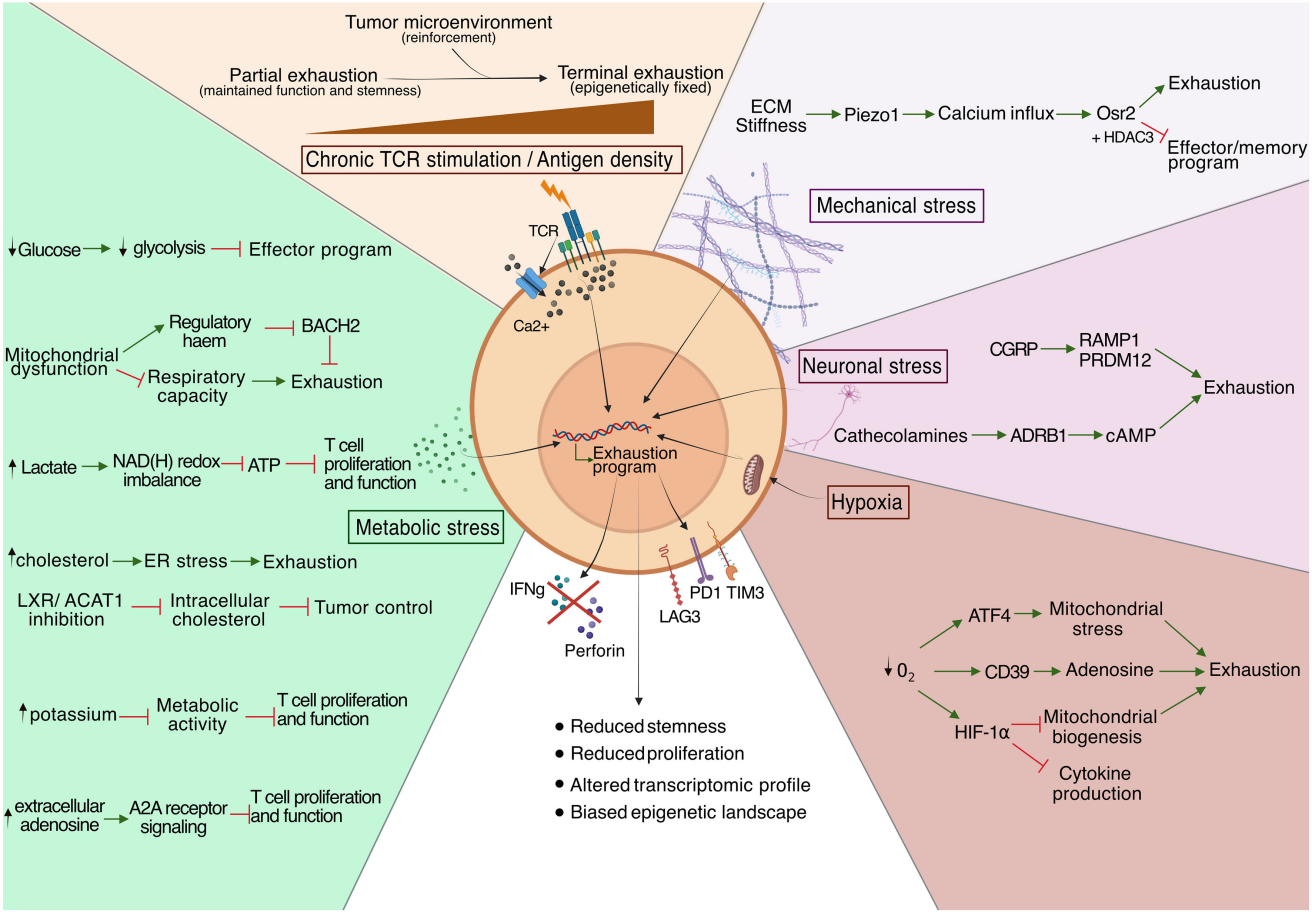
Pathways enforcing tumor-induced CD8^+^ T cell exhaustion in the TME. Multiple pathways within the tumor microenvironment (TME) enforce CD8^+^ T cell exhaustion and create a tumor-specific suppressive niche. Chronic and strong TCR stimulation, driven by high antigen density, promotes progression toward terminal exhaustion, impairing effector function and stemness of CD8^+^ TILs. Tumor architecture further shapes T cell differentiation through mechanosensory signaling: extracellular matrix (ECM) stiffness activates the Piezo1–Osr2 axis, inducing exhaustion-associated gene transcription and HDAC3-dependent repression of effector and memory programs. Hypoxia limits T cell function via HIF-1α and ATF4, impairing mitochondrial biogenesis, inducing stress responses, and reducing cytokine production. It also upregulates CD39 on T cells and T_REG_, increasing extracellular adenosine and reinforcing exhaustion. The TME is also metabolically restrictive: glucose deprivation suppresses glycolysis and cytokine production, while mitochondrial dysfunction reduces respiratory capacity and increases proteasomal activity, elevating regulatory haem levels that inhibit BACH2 and promote terminal exhaustion. Elevated lactate disrupts NAD(H) redox balance, and cholesterol imbalance induces ER stress or impairs tumor control, highlighting complex metabolic regulation. Additional factors such as high extracellular potassium from necrosis and adenosine signaling via A2A receptors further suppress proliferation and effector functions. Collectively, these tumor-imposed stresses converge on transcriptional and epigenetic programs that reduce proliferation and stemness, increase inhibitory receptor expression, and lock CD8^+^ T cells into a dysfunctional, exhausted state.

Tumor hypoxia (**Figure 1**) represents a central environmental stress that profoundly shapes CD8^+^ T cell differentiation and function within the tumor microenvironment (TME). Mechanistically, low oxygen tension stabilizes hypoxia-inducible factor-1α (HIF-1α), reprogramming T cells through transcriptional rewiring, metabolic restriction, and altered effector differentiation. Early studies demonstrated that HIF-1α expression in tumor-associated macrophages suppresses T cell activity and promotes tumor progression, highlighting hypoxia as a potent extrinsic modulator of antitumor immunity (32). Subsequent work revealed that intratumoral hypoxia restricts mitochondrial biogenesis in CD8^+^ T cells, causing metabolic insufficiency that predisposes them to dysfunction (33). Complementary studies confirmed that HIF-1α acts intrinsically within CD8^+^ T cells to shape their effector program, further linking oxygen deprivation to impaired antitumor responses (34).

Beyond these foundational insights, recent investigations have clarified the mechanisms by which hypoxia drives terminal T cell exhaustion. Continuous T cell stimulation under low oxygen rapidly induces mitochondrial stress, reinforcing a transcriptional and metabolic state characteristic of exhausted T cells (35). Hypoxia further supports the differentiation of terminally exhausted CD8^+^ T cells and promotes the acquisition of suppressive pathways, such as CD39-dependent adenosinergic activity, that limit antitumor immunity (36, 37). Notably, chronic hypoxic stress activates integrated stress response pathways, including ATF4, which consolidate T cell dysfunction within tumors (38). Proteomic analyses corroborate these findings, showing that hypoxia dominantly remodels the effector T cell surface landscape, influencing both activation status and sensitivity to regulatory T cell-mediated suppression (39). Collectively, these studies underscore hypoxia as a central driver of T cell exhaustion in the TME, integrating metabolic, transcriptional, and functional pathways. Understanding this axis provides a conceptual framework for targeting hypoxia-induced vulnerabilities in exhausted T cells to enhance antitumor immunity.

### 2.2 Mechanical stress in the tumor microenvironment

The tumor microenvironment is not only chemically hostile but also biophysically abnormal. Solid tumors develop extensive extracellular matrix (ECM) remodeling (**Figure 1**), leading to elevated stiffness, compressive and tensile forces, and altered mechanical stress profiles that can directly impair immune cell function (40). Dense collagen deposition, crosslinking, and activation of cancer-associated fibroblasts all contribute to increased ECM stiffness, which acts as both a physical barrier to immune cell infiltration and an active biomechanical cue that alters leukocyte behavior and phenotype (41). Stiffened matrices impede T cell trafficking into tumor cores and mechanically activate mechanotransduction pathways that suppress effector functions and immune surveillance mechanisms. Elevated matrix stiffness and associated solid stresses compress the extracellular space, reduce immune cell motility, and disrupt tertiary lymphoid structures critical for sustained antitumor responses. Mechanotransduction signaling in response to altered rigidity and stress regulates immune cell migratory capacity, activation thresholds, and effector gene expression, cumulatively contributing to an immunosuppressive TME (42).

Recent work by Zhang et al. identified a direct molecular link between tumor mechanical stress and CD8^+^ T cell exhaustion through a biomechanical checkpoint involving the transcription factor Osr2. In this study, solid tumor ECM stiffness and mechanical stress signaling via the mechanosensitive receptor Piezo1 and downstream CaMKII/CREB axis induced Osr2 selectively in terminally exhausted tumor-specific CD8^+^ T cells. Osr2 functions as a mechanosignaling integrator that exacerbates exhaustion: it recruits histone deacetylase 3 (HDAC3) to remodel chromatin and suppress cytotoxic effector gene programs, increasing expression of exhaustion markers such as PD-1 and TIM-3 and diminishing cytokine production. Deletion of Osr2 in T cells alleviated mechanical stress-driven exhaustion and improved antitumor efficacy of both naive and CAR-T cells in solid tumor models, whereas forced Osr2 expression worsened dysfunction. These findings establish Osr2 as a biomechanical checkpoint that links the altered physical forces of the TME with epigenetic and transcriptional programs driving CD8^+^ T cell exhaustion, and suggest that targeting mechanotransduction pathways may enhance immunotherapeutic responses in stiff tumor niches (43, 44).

### 2.3 Tumor-specific metabolic environment

Tumor metabolism (**Figure 1**) profoundly shapes T cell function and the efficacy of immunotherapies. Altered nutrient availability, accumulation of inhibitory metabolites, and ionic imbalances within the tumor microenvironment (TME) impair T cell proliferation, differentiation, and cytotoxicity. Understanding and manipulating these metabolic interactions is crucial for optimizing immunotherapy outcomes (45, 46).

#### 2.3.1 Glucose restriction

Tumor and immune cells actively compete for nutrient within the TME, creating a state of metabolic competition that profoundly affects antitumor immunity. Because highly glycolytic tumor cells consume large amounts of glucose, tumor-infiltrating CD8^+^ T cells (TILs) frequently experience nutrient deprivation, leading to reduce glycolytic activity, impaired cytokine production and diminished effector function. It was already demonstrated that this metabolic competition directly contributes to T cell dysfunction and promotes tumor progression (47). *Ho* et al. identified phosphoenolpyruvate (PEP) as a critical metabolic checkpoint regulating T cell effector response. Reduced glycolytic flux in glucose-restricted T cells decreased intracellular PEP levels, thereby impairing Ca^2+^-NFAT signaling and limiting antitumor activity (48). Enhancing glucose uptake in T cells, for instance by restoring PEP production (48) or through Glut3 overexpression (49), can restore energy metabolism and improve antitumor efficacy in solid tumors. Moreover, metabolic exhaustion, including cholesterol deficiency, exacerbates T cell dysfunction, further limiting antitumor immunity (50). Strategies that augment T cell nutrient acquisition or bypass tumor-induced glucose restrictions hold promise for improving CAR-T and other adoptive cell therapies.

#### 2.3.2 Mitochondrial fitness

Within the TME, tumor infiltrating lymphocytes also exhibit disturbed mitochondrial dynamics. Yu et al. demonstrated that altered mitochondrial fission/fusion balance in CD8^+^ TILs reinforces exhaustion programs by limiting respiratory capacity and sustaining inhibitory receptor expression. Further research revealed that the accumulation of depolarized mitochondria in CD8^+^ TILs pushes exhaustion and limits stemness through enhanced proteasomal activity, resulting in increased regulatory haem levels (RH). RH directly regulates the transcription factor BACH2, rewiring the transcriptional network and promoting terminal exhaustion of CD8^+^ TILs (51). These findings position mitochondrial architecture itself as a structural regulator of exhaustion stability within tumors (52). Interestingly, Guo et al. demonstrated that IL-10–mediated metabolic reprogramming can partially restore mitochondrial metabolism in terminally exhausted CD8^+^ T cells, enhancing antitumor immunity. This study challenges the view of IL-10 as purely suppressive and highlights how cytokine-driven metabolic rewiring can counteract tumor-imposed dysfunction, even in deeply exhausted populations (53).

#### 2.3.3 Lactate accumulation

Rapidly proliferating tumor cells produce large amounts of lactate, acidifying the TME. Acidic lactate-rich environment impairs T cell proliferation and effector function by perturbing the NAD(H) redox balance, restricting ATP generation and biosynthetic capacity thereby promoting exhaustion program and limiting response to checkpoint blockade (54). However, the respective contribution of lactate versus extracellular acidosis to T cell dysfunction remain difficult to disentangle *in vivo*. It has been demonstrated that intratumoral acidity leads to impairment of the T cell response and cytotoxicity (55). Further studies showed that activated T cells can also import lactate and use it as a metabolic substrate to fuel Krebs cycle under specific metabolic conditions. Indeed, it was suggested that pH-neutral lactate supplementation enhanced CD8^+^ T cells cytotoxicity in both nutrient-replete and nutrient-deprived environments (56, 57). These findings highlight the context-dependent role of lactate metabolism within the TME and suggest that therapeutic strategies targeting lactate production, transport or buffering must carefully consider both its immunosuppressive and metabolic functions.

#### 2.3.4 Cholesterol and lipid metabolism

Tumor-derived cholesterol has been reported to trigger ER stress in CD8^+^ T cells, leading to upregulation of inhibitory receptors and functional exhaustion (58). However, other studies provided evidence for cholesterol insufficiency in the intratumoral T cell compartment. Ablation of the LXR transcription factor, transcribing genes for cholesterol efflux and inhibition of cholesterol uptake, in CAR-T cells resulted in heightened tumor control capacity. Similarly, ablation and pharmacological inhibition of ACAT1, a key cholesterol esterification enzyme, in CD8^+^ T cells ameliorated their anti-tumor efficacy (59). More recently, it has been demonstrated that exhaustion-associated cholesterol deficiency directly impairs the cytotoxic program of CD8^+^ T cells, further highlighting the dual and context-dependent role of cholesterol imbalance within the TME (50). These findings underscore the complexity of the cholesterol imbalance within the TME. Beyond cholesterol, altered lipid metabolism can influence T cell membrane composition and signaling, impacting immune synapse formation and cytokine secretion. The different specific lipids regulating CD8^+^ T cell function within the tumor have been reviewed elsewhere (60). Modulating lipid availability or cellular cholesterol handling may therefore reinvigorate T cells and improve immunotherapy efficacy (50, 58).

#### 2.3.5 Ionic imbalance

Necrotic tumor regions release high levels of potassium, creating an ionic microenvironment that suppresses T cell activation and effector gene expression (61). This ionic immune checkpoint reduces T cell metabolic activity and limits proliferation, representing a noncanonical mechanism of immune evasion. Therapeutic approaches that restore ionic homeostasis or enhance T cell resilience to potassium stress could potentiate antitumor responses (62). Recently, magnesium has emerged as an important regulator of CD8^+^ T cell activation and effector function. Lötscher et al. demonstrated that extracellular Mg^2+^ is required for LFA-1 activation, promoting calcium signaling, immune synapse formation and metabolic reprogramming resulting in efficient cytotoxic CD8^+^ T cell activity. Conversely, magnesium deficiency impaired anti-tumor T cell response and reduced the efficacy of immunotherapies (63). These findings highlight the critical link between ionic homeostasis and immune cell functionality and further emphasize the therapeutic relevance of the tumor ionic microenvironment.

#### 2.3.6 Adenosine accumulation

Extracellular adenosine, generated via ectonucleotidases such as CD39 and CD73, suppresses T cell function through the A2A receptor signaling axis, impairing proliferation, cytokine production, and cytotoxicity. CD39 is expressed on regulatory T cells (T_REG_) and on CD8^+^ TILs themselves, which forms a positive feedback loop stabilizing exhaustion and a layer of T_REG_-mediated suppression. Blocking adenosine signaling or enzymatic production enhances T cell infiltration and function within tumors, providing a complementary strategy to checkpoint inhibition (64).

In summary, tumor-specific metabolic cues, including nutrient scarcity, lactate, cholesterol, potassium, and adenosine, collectively shape T cell functionality and the outcome of immunotherapies. Rational metabolic engineering of T cells or targeted modulation of the TME offers promising avenues to overcome these suppressive mechanisms and enhance the efficacy of CD8^+^ T cell–based cancer therapies (65).

### 2.4 Implication of the neural system

Tumor, neural system and immune system form a complex communication axis within the TME. The neural system can enforce T cell exhaustion through the activation of the β1-adrenergic receptor ADRB1 on CD8^+^ T (66). This is particularly relevant for cancer patients who display increased stress and increased levels of circulating catecholamines (67). Other knock outs (KO), such as calcitonin gene-related peptide (CGRP) complete KO, display reduced tumor growth, increased immune infiltration and increased cytotoxic CD8^+^ T cells, without a direct link to T cell exhaustion (68). A more recent study provided further mechanistic insights on how CGRP shapes antitumor activity of TILs. CGRP is sensed by CD8^+^ TILs through a receptor complex comprising RAMP1 which activates the transcription factor PRDM12, leading to epigenetic remodeling and suppression of effector genes (69). These new insights into the neuroimmune crosstalk provide the rationale to investigate additional pathways that might emerge in the regulation of exhaustion.

### 2.5 Chronic antigen density and TCR signaling strength in tumors

Chronic antigen exposure and TCR signaling strength are central determinants of T cell fate in tumors (**Figure 1**). While persistent antigen recognition and TCR engagement are also hallmarks of chronic infections, tumor-specific contexts impose additional layers of regulation that distinguish cancer immunity. In tumors, T cells encounter self- or neoantigens in the context of immunosuppressive cues, metabolic restrictions, and spatial confinement, which collectively drive progressive dysfunction without deletion, demonstrating how tumor-specific chronic stimulation shapes differentiation (4). Epigenetic fixation under these conditions locks T cells into dysfunctional states, limiting their proliferative capacity and cytotoxic potential, and differentiating tumor-exhausted T cells from those observed in viral infections.

Recent studies reveal that the density and persistence of antigen, together with TCR signal intensity, orchestrate distinct T cell programs in solid tumors. Chronic antigen exposure promotes a unique tissue-resident phenotype, giving rise to resident memory-like or exhausted T cell subsets with specialized transcriptional and functional profiles (70, 71). These resident populations are further shaped by tumor-specific metabolic and immunosuppressive environments, which reinforce dysfunction and constrain effector activity, a feature less pronounced in infection-driven T cell exhaustion. TCR signal strength modulates the mechanisms of exhaustion. High-intensity signaling induces terminally exhausted T cells with stable epigenetic programs, whereas lower-intensity or intermittent signals generate partially exhausted cells capable of limited effector function (72). This graded response highlights that, in tumors, TCR engagement interacts with local environmental factors to define the magnitude and reversibility of dysfunction, beyond the simpler models derived from chronic infection.

In summary, chronic antigen exposure and TCR signal strength define T cell differentiation in tumors, but the interplay with tumor-specific factors creates unique patterns of dysfunction. These insights provide a framework for interventions aimed at reversing exhaustion and improving immunotherapy outcomes. Therapeutically, understanding these tumor-specific distinctions informs the design of adoptive cell therapies, checkpoint blockade, and neoantigen vaccines. Engineering T cells to resist epigenetic fixation, or modulating antigen presentation, could preserve effector function and enhance persistence. Fine-tuning TCR signal intensity through receptor affinity or co-stimulation provides additional avenues to reshape T cell differentiation toward sustained anti-tumor activity.

Building on these insights, it becomes clear that the extrinsic cues of the tumor microenvironment, e.g. hypoxia, mechanical stress, metabolic competition, and chronic antigen stimulation, not only induce T cell dysfunction but also actively reinforce and stabilize it. These environmental signals intersect with T cell-intrinsic programs, consolidating exhaustion through durable transcriptional and epigenetic remodeling. In other words, the tumor does not merely suppress T cell function transiently; it shapes a stable, self-sustaining exhausted state that is encoded at the level of gene expression and chromatin architecture. Understanding how these tumor-specific cues are translated into persistent transcriptional and epigenetic programs is therefore essential for unraveling the mechanisms that limit antitumor immunity and for designing strategies to reverse exhaustion. The next section will focus on these tumor-reinforced transcriptional and epigenetic landscapes, highlighting how the TME sculpts durable T cell dysfunction and identifying potential avenues to therapeutically reprogram exhausted CD8^+^ T cells.

## 3 Tumor-reinforced transcriptional and epigenetic programs of exhausted CD8 T cells

### 3.1 The NFAT family

**Figure 2:**
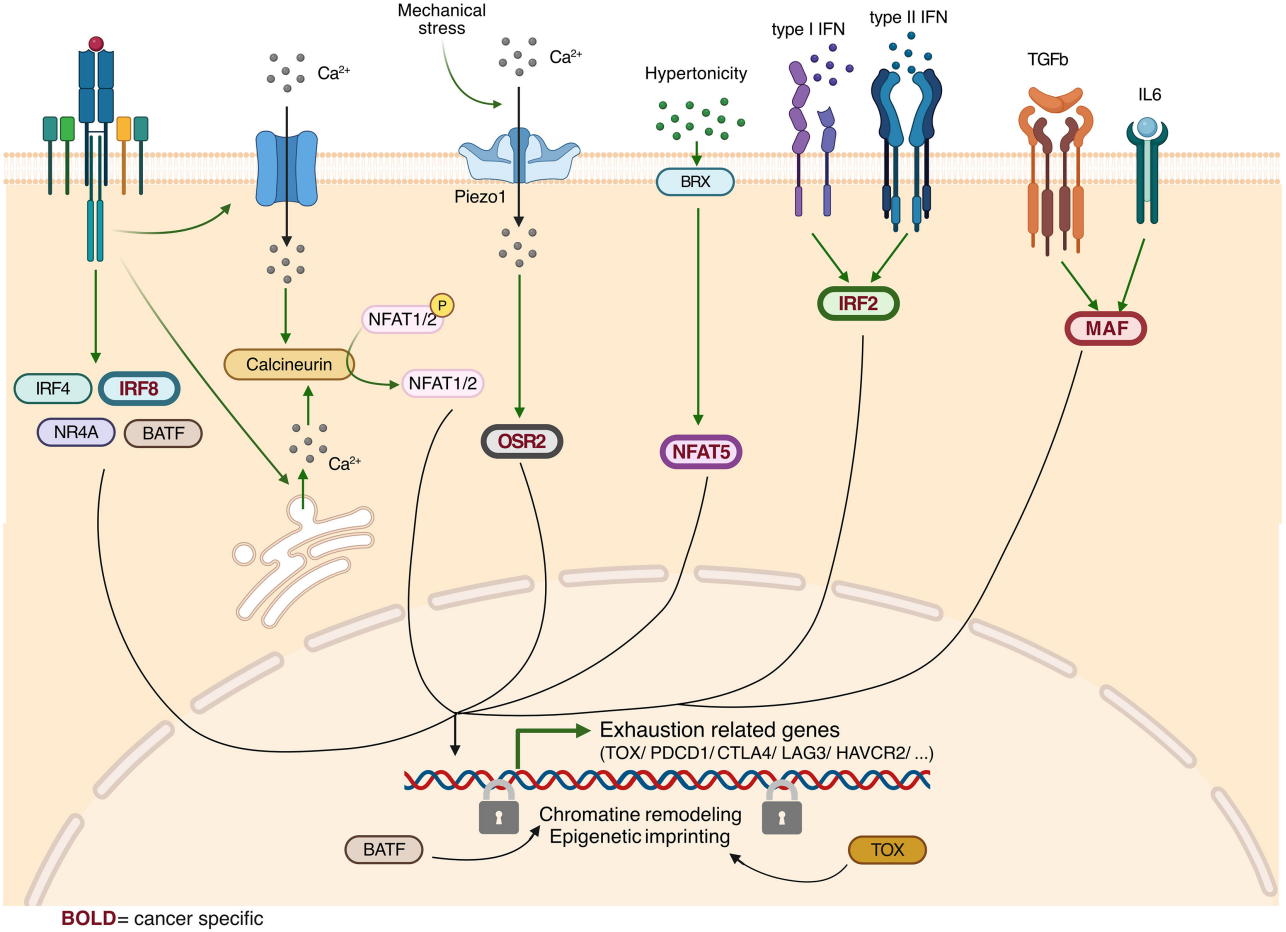
Transcriptomic and epigenetic regulation in tumor-induced CD8^+^ T cell exhaustion. CD8^+^ T cell–intrinsic pathways drive exhaustion in tumors (cancer-associated transcription factors are shown in bold). Both acute and chronic TCR stimulation induce NFAT, but chronic signaling reduces AP-1 activity, leading to an “NFAT without AP-1” program characterized by increased inhibitory receptor expression and reduced effector and memory gene expression. Additional TCR-induced factors, including IRF4, IRF8, NR4A family members (NR4A1–3) and BATF, reinforce exhaustion through direct transcriptional regulation and chromatin remodelling. Tumor microenvironmental cues further sustain this program. Mechanical stress sensed by Piezo1 activates OSR2 promoting expression of exhaustion associated genes. Hypertonicity, notably high extracellular potassium, activates NFAT5, impairing effector function and promoting inhibitory receptor expression. Chronic inflammatory signaling induces regulators such as IRF2, while TGF-β and IL-6 promote MAF expression, driving terminal differentiation and limiting anti-tumor activity. In parallel, factors such as BATF and TOX reshape chromatin accessibility, reinforcing exhaustion-associated programs over effector and memory states. Together, these transcriptional and epigenetic networks converge to establish and stabilize the exhausted phenotype in CD8^+^ TILs.

As mentioned previously, chronic TCR stimulation induces NFAT1/2-dependent transcriptional rewiring (**Figure 2**) (13). Further mechanistic resolution identified TOX and NR4A1–3 as critical downstream targets of chronic NFAT1/2 signaling (22). Persistent NFAT activation induces TOX expression, which in turn orchestrates widespread epigenetic remodeling and stabilizes the exhausted state (14). This pathway forms the transcriptional backbone through which chronic antigen exposure in tumors and chronic infection is translated into durable exhaustion programs.

Recent work has expanded the role of the NFAT family in this process by implicating NFAT5, a tonicity-responsive transcription factor distinct from calcineurin-dependent NFAT family members (**Figure 2**). Unlike NFAT1–4, NFAT5 responds to osmotic and ionic stress rather than calcium/calcineurin signaling (73). Although initially characterized in the context of hypertonic stress, NFAT5 is increasingly relevant to tumors, where ionic imbalance and metabolic stress are prominent features of the microenvironment. NFAT5 is essential for T cell survival under hypertonic conditions establishing its role as a stress-adaptive regulator (74). These findings gain particular significance when integrated with tumor-specific ionic dysregulation. Elevated extracellular potassium in tumors suppresses T cell effector gene expression and limits cytotoxicity (75), creating osmotic and ionic conditions that may engage NFAT5-dependent pathways. Thus, in addition to calcineurin-driven NFAT activation downstream of chronic TCR signaling, the tumor microenvironment may reinforce dysfunction through NFAT5-mediated stress sensing. Importantly, a recent study provided direct evidence that NFAT5 is not merely a stress-adaptive factor but an active enforcer of CD8^+^ T cell exhaustion within tumors. The authors demonstrated that NFAT5 becomes selectively activated in tumor-infiltrating CD8^+^ T cells in response to microenvironmental stress signals, including ionic and osmotic perturbations. Genetic ablation of NFAT5 in T cells attenuated the acquisition of exhaustion markers, restored effector gene expression, and improved antitumor activity, establishing a causal role for NFAT5 in reinforcing dysfunction. Mechanistically, NFAT5 integrated environmental stress cues into a transcriptional program overlapping with terminal exhaustion signatures, promoting inhibitory receptor expression while constraining cytotoxic and metabolic pathways. These findings position NFAT5 as a critical node that translates tumor-imposed stress into durable transcriptional remodeling (76).

Taken together, those studies expand the classical NFAT–TOX axis driven by chronic TCR signaling. Whereas calcineurin-dependent NFAT activation links persistent antigen stimulation to exhaustion, NFAT5 provides a parallel, stress-responsive pathway that amplifies and stabilizes this state under tumor-specific conditions. Thus, exhaustion in cancer emerges not solely from sustained antigen engagement but from the convergence of chronic TCR signaling and microenvironmental stress at the level of NFAT family transcriptional control. This dual NFAT circuitry may explain why tumor-exhausted T cells exhibit particularly stable and refractory dysfunction compared to exhaustion observed in other chronic settings.

### 3.2 The IRF family

Among transcriptional regulators shaping CD8^+^ T cell differentiation, members of the interferon regulatory factor (IRF) family, represent critical yet often underappreciated nodes in tumor-associated exhaustion (**Figure 2**). Several members have been associated specifically to T cell exhaustion during chronic infection or cancer. IRF4 and IRF8 have been shown to be regulated by TCR engagement in CD8^+^ T cells, thus their functions are highly relevant to cancer because they interpret TCR signal strength, integrate metabolic cues, and govern lineage stability under persistent stimulation. Tumors exploit these properties to skew differentiation toward dysfunctional states.

#### 3.2.1 IRF4: a signal strength interpreter in tumors

IRF4 functions as a quantitative interpreter of TCR signaling. It was first demonstrated that IRF4 expression scales proportionally with TCR/BCR signal intensity, determining its partner as well as binding sites, therefore establishing it as a molecular rheostat linking antigen engagement to transcriptional output (62). Then, it was further shown that IRF4 regulates effector differentiation through metabolic programming, coupling TCR strength to glycolytic and biosynthetic pathways necessary for cytotoxic function (77, 78). Thus, IRF4 integrates signaling intensity with metabolic capacity, a feature particularly relevant in the nutrient-restricted tumor microenvironment. Under conditions of persistent antigen exposure, IRF4 contributes to shaping differentiation trajectories. It has been shown that sustained IRF4 activity influences CD8^+^ T cell fate decisions during chronic stimulation (79). In chronic infection models, IRF4 promotes exhaustion and limits the development of memory-like T cells (80), indicating that prolonged high-level IRF4 expression reinforces dysfunctional differentiation programs.

In tumors, these mechanisms acquire additional specificity. Chronic antigen density, sustained TCR engagement, and metabolic stress create conditions that maintain elevated IRF4 activity. However, unlike infection, tumor T cells operate within hypoxic, nutrient-deprived, and mechanically stressed environments. These contextual pressures amplify the consequences of IRF4-driven programming. Notably, Seo et al. demonstrated that BATF and IRF4 cooperate to counter exhaustion in tumor-infiltrating CAR-T cells. This study revealed that the IRF4/BATF axis can rewire chromatin accessibility and sustain effector programs when properly modulated, suggesting that IRF4 is not merely exhaustion-promoting but context-dependent. In tumors, insufficient or dysregulated IRF4/BATF activity may tip the balance toward exhaustion rather than durable effector function (81).

Thus, IRF4 emerges as a tumor-sensitive interpreter of persistent TCR signals: its sustained expression under chronic stimulation contributes to exhaustion trajectories, yet its interaction partners and metabolic context determine whether it reinforces dysfunction or preserves antitumor potency. Tumors exploit this signal strength–dependent plasticity to bias differentiation toward terminal states.

#### 3.2.2 IRF8: a balance for effector vs exhaustion differentiation in cancer

IRF8 plays a complementary but distinct role in regulating CD8^+^ T cell fate. It was first demonstrated that IRF8 is essential for effector differentiation and memory formation, stabilizing cytotoxic gene programs and supporting lineage commitment. IRF8 deficiency skews differentiation trajectories and destabilizes effector programs features that become highly relevant in tumor settings where differentiation bias is a defining characteristic of T cell dysfunction (82). Later work done using targeted IRF8 deletion in T cells reached a contrasting conclusion underlining a role for IRF8 as negative regulator of CD8^+^ T cell differentiation. The authors showed that in a herpes simplex virus infection model, CD8^+^ T cell lacking IRF8 had greater expansion capacity associated with stronger effector functions, suggesting a more complex role for IRF8 in CD8^+^ T cells than initially thought (83).

Although early work primarily examined infection models, emerging evidence underscores tumor-specific roles for IRF8. It was showed that tumor-associated macrophages expressing IRF8 promote CD8^+^ T cell exhaustion in cancer. This finding is particularly important because it shifts the perspective from T cell-intrinsic regulation to microenvironmental orchestration. In tumors, IRF8 activity within myeloid compartments shapes cytokine landscapes and antigen presentation contexts that drive exhaustion programs in T cells. Thus, IRF8 functions not only within CD8^+^ T cells but also as a regulator of the broader exhaustion-permissive niche (84). Recently, Li et al. provided mechanistic insight by analyzing the three-dimensional genome architecture of exhausted CD8^+^ T cells in cancer. Their study revealed that IRF8 plays a critical role in organizing chromatin topology associated with exhaustion states. Altered IRF8-dependent regulatory networks were shown to influence enhancer/promoter interactions governing effector and inhibitory gene expression. These findings position IRF8 as a structural regulator of exhaustion-associated genome organization, directly linking tumor-driven differentiation bias to stable epigenetic remodeling (85). Consistent with these observations, Ongaro et al. identified IRF8 as a tumor-specific regulator of CD8^+^ T cell exhaustion. IRF8 expression was selectively induced in TILs but not in chronic infection, in which it promoted exhaustion by directly supporting TOX expression. Conversely, IRF8 KO in T cells resulted in enhanced effector cytokine production, reduced exhaustion-associated features and improved tumor control (86).

Importantly, the tumor context imposes persistent antigen stimulation alongside hypoxia, metabolic stress, and inflammatory cytokine gradients. These combined cues can distort IRF8-mediated differentiation programs, shifting CD8^+^ T cells away from durable effector or memory states and toward exhaustion. Unlike infection, where antigen clearance eventually relieves differentiation pressure, tumors maintain continuous signaling and environmental stress, reinforcing IRF8-dependent exhaustion architecture. Being the closest structural and functional homologs, with many shared transcriptional partners, IRF4 and IRF8 form a transcriptional axis that interprets chronic TCR signaling and stabilizes lineage decisions. In tumors, persistent antigen density sustains IRF4 expression, while environmental stressors and myeloid-derived signals modulate IRF8-dependent chromatin landscapes. The convergence of these factors drives differentiation toward terminally exhausted states characterized by stable epigenetic fixation and diminished plasticity. Beyond IRF4 and IRF8, additional IRF family members contribute to CD8^+^ T cell differentiation in cancer, most notably IRF2.

#### 3.2.3 IRF2: an enforcer of interferon-mediated exhaustion in cancer

Recent work demonstrated that IRF2 drives interferon-mediated CD8^+^ T cell exhaustion within tumors, thereby restricting antitumor immunity. In this study, IRF2 was shown to integrate chronic type I interferon signaling, variably present in different tumor microenvironments, into a transcriptional program that reinforced inhibitory receptor expression and limited effector function. Genetic disruption of IRF2 in CD8^+^ T cells reduced exhaustion features and improved tumor control, establishing a tumor-intrinsic role for IRF2 in stabilizing dysfunctional states (87). Importantly, this identifies a mechanistic link between sustained interferon exposure in cancer and exhaustion-specific transcriptional reinforcement, distinguishing tumor settings from acute antiviral responses where interferon signaling is transient and protective.

#### 3.2.4 Other IRFs to investigate

In contrast, IRF5, IRF7 and IRF9 have been primarily characterized in viral infection models. IRF9 prevents CD8^+^ T cell exhaustion in an extrinsic manner during acute lymphocytic choriomeningitis virus (LCMV) infection (88), while IRF5 regulates lipid metabolism and mitochondrial function in CD8^+^ T cells during viral responses (89). IRF7 has been shown to promote terminal differentiation of virus-specific CD8^+^ T cells during chronic LCMV infection (90). As for IRF2 in the context of tumors, IRF7 drives exhaustion as a consequence of prolonged type I IFN signaling, viewed as detrimental to T cell-mediated immunity, thus integrating a further chronic stimulus into fate decision and differentiation. A further member of the IRF family associated to CD8^+^ T cell differentiation in tumors is IRF1. Zhou et al. identified RBPJ through a single-cell CRISPR/Cas9-based screening as an important factor inhibiting IRF1 in intermediate exhausted T cells, allowing differentiation toward terminal exhaustion. Their results underscore a rather positive role for IRF1 in CD8^+^ T cell function within tumors (91).

Collectively, these findings highlight that although IRF family members are broadly involved in T cell differentiation across infectious and inflammatory contexts, tumors uniquely co-opt specific IRF-dependent pathways to enforce exhaustion. While IRF5, IRF7 and IRF9 predominantly regulate antiviral immunity and metabolic fitness in infection settings, IRF2, IRF4, and IRF8 acquire tumor-specific relevance under conditions of persistent antigen exposure, chronic interferon signaling, and microenvironmental stress. In cancer, sustained TCR engagement maintains IRF4 activity as a signal-strength interpreter, IRF8 shapes exhaustion-associated chromatin architecture and differentiation bias, and IRF2 translates prolonged interferon exposure into stable dysfunctional programs. Chronic stimulation without antigen resolution, combined with metabolic restriction, hypoxia, and inflammatory cues, skews IRF-driven transcriptional networks toward terminal exhaustion rather than memory or durable effector states. Thus, the tumor microenvironment does not merely activate IRF pathways, it repurposes their signal-sensing and chromatin-organizing functions to stabilize CD8^+^ T cell dysfunction. Therapeutically, targeting IRF-centered regulatory circuits, whether by recalibrating TCR signal interpretation (IRF4), restoring effector chromatin topology (IRF8), or modulating interferon-driven exhaustion programs (IRF2), may provide strategic avenues to reprogram tumor-infiltrating T cells and restore antitumor immunity.

### 3.3 BATF-driven chromatin remodeling in tumor

Basic Leucine Zipper ATF-Like Transcription Factor (BATF) is a central regulator of chromatin remodeling in exhausted CD8^+^ T cells. As a member of the AP-1 transcription factor family, BATF functions not merely as a downstream effector of TCR signaling, but as a lineage-defining factor that reshapes chromatin accessibility and stabilizes differentiation programs under chronic stimulation (**Figure 2**). Its role becomes particularly critical in tumors, where persistent antigen exposure and microenvironmental stress converge to lock T cells into dysfunctional states.

BATF is required for CD8^+^ T cell differentiation during chronic antigen exposure. It was demonstrated that BATF regulates the transition from progenitor-like to cytolytic effector CD8^+^ T cells during chronic viral infection, positioning BATF as a key interpreter of sustained TCR signals. Rather than simply activating effector genes, BATF shapes the broader differentiation trajectory of CD8^+^ T cells exposed to persistent antigen. This establishes BATF as a transcriptional rheostat that calibrates responses when antigen is not cleared as observed in cancer (92). Under chronic conditions, BATF does not act alone. It cooperates with IRF4 to form composite AP-1/IRF complexes that bind specific regulatory elements. These complexes are particularly enriched at exhaustion-associated loci, suggesting that BATF is not only involved in differentiation but in the acquisition of dysfunctional programs. BATF functions as a pioneer-like factor capable of establishing accessible chromatin regions that guide CD8^+^ T cell differentiation. During chronic viral infection, BATF is required for the transition from progenitor-like states toward cytolytic effector differentiation, as demonstrated by Chen et al., who showed that BATF deficiency disrupts the acquisition of effector programs under persistent antigen stimulation, underscoring its importance in sustaining T cell responses when antigen clearance fails (12).

Mechanistically, chronic TCR signaling activates NFAT1/2, which induces IRF4 expression. IRF4 then cooperates with BATF to bind composite AP-1/IRF elements (AICEs), promoting accessibility at key regulatory loci. This NFAT-IRF4-BATF axis translates signal strength and persistence into epigenetic remodeling. Importantly, this partnership does not merely activate effector genes but progressively shapes a chromatin architecture compatible with exhaustion (81). The pioneer role of BATF in effector differentiation has also been highlighted by Fujisawa et al., who demonstrated that BATF interacts with IRF4 to initiate the differentiation program of effector CD8^+^ T cells (93). Thus, BATF sits at a critical bifurcation point: it enables effector specification but, under chronic stimulation, contributes to the transition toward dysfunctional states.

Although BATF also operates in chronic viral infection, tumors uniquely harness BATF-driven chromatin remodeling to stabilize dysfunction within a metabolically hostile and immunosuppressive microenvironment. Evidence from CAR-T cell studies provides compelling support for a tumor-specific dimension. Zhang et al. demonstrated that depletion of BATF in CAR-T cells enhances antitumor activity, promotes central memory formation, and confers resistance to exhaustion. BATF-deficient CAR-T cells exhibit improved persistence and functionality in tumor settings, indicating that BATF contributes directly to the stabilization of exhaustion programs in cancer (94). Mechanistically, the tumor microenvironment amplifies chronic TCR signaling while imposing metabolic stress, hypoxia, and suppressive cytokine exposure. Under these conditions, sustained NFAT1/2 activation reinforces IRF4 and BATF cooperation, consolidating exhaustion-specific chromatin accessibility. Recent work targeting the BATF2/RGS2 axis further underscores this tumor-specific adaptation. Gu et al. showed that disrupting this pathway reduces T cell exhaustion and restores antitumor immunity, suggesting that BATF-related transcriptional modules integrate tumor-derived signals to reinforce inhibitory gene expression and limit effector resilience (95).

### 3.4 NR4A tumor-specific reinforcement layer of exhaustion

The NR4A family of orphan nuclear receptors (NR4A1, NR4A2, and NR4A3) functions as a critical reinforcement module downstream of chronic TCR signaling, consolidating transcriptional and metabolic programs associated with CD8^+^ T cell dysfunction (**Figure 2**). While NR4A factors are induced in settings of persistent antigen stimulation more broadly, their role in cancer is distinguished by their capacity to stabilize tumor-specific exhaustion and limit durable antitumor immunity (96).

First, it was demonstrated that NR4A transcription factors directly restrain effector differentiation and enforce dysfunctional gene programs in tumor-infiltrating CD8^+^ T cells. NR4A proteins are induced by sustained NFAT signaling under chronic TCR engagement, where they bind exhaustion-associated enhancers and promote accessibility at loci encoding inhibitory receptors and negative regulators of effector function. Importantly, combined deletion of NR4A family members reprogrammed tumor-specific T cells toward enhanced cytokine production, increased cytotoxicity, and superior tumor control. In CAR-T cell models, NR4A-deficient cells exhibited markedly improved antitumor efficacy, providing direct tumor-context evidence that NR4A acts as an active enforcer, not merely a marker, of dysfunction (22). Mechanistically, NR4A factors cooperate with other exhaustion-associated transcription factors, including TOX and AP-1 family members, to reinforce chromatin landscapes initiated by chronic signaling. In this context, NR4A does not simply dampen effector responses, it contributes to locking T cells into a transcriptionally restrained, metabolically compromised state that favors tolerance over clearance (35, 93, 96).

Recent translational work further highlights this tumor-specific dimension. Nakagawara et al. showed that NR4A ablation in human CAR-T cells improves mitochondrial fitness, enhances metabolic resilience, and supports long-term persistence in solid tumor models. NR4A-deficient CAR-T cells displayed superior oxidative metabolism and sustained effector capacity, directly linking NR4A activity to the metabolic fragility characteristic of tumor-infiltrating T cells. Thus, beyond transcriptional repression, NR4A integrates chronic signaling with mitochondrial dysfunction, an axis particularly relevant in the nutrient-restricted tumor microenvironment (97).

Collectively, these findings position the NR4A family as a tumor-amplified reinforcement layer of exhaustion. While NR4A factors are inducible in any chronic stimulation context, tumors uniquely exploit their capacity to consolidate inhibitory transcriptional programs and impose metabolic constraints. Targeting NR4A-centered networks therefore represents a strategy not only to relieve transcriptional repression but also to restore metabolic competence and persistence in tumor-reactive CD8^+^ T cells.

### 3.5 MAF integrates tumor-derived environmental signals

The regulatory network governing CD8^+^ T cell differentiation and dysfunction is broad and context-dependent, influenced by microenvironmental factors that in tumors are highly relevant. In addition to the actors described above, the transcription factor MAF has been shown to be particularly expressed in TILs, promoting a tumor-associated dysfunctional program in CD8^+^ T cells (**Figure 2**). While MAF KO in tumor-specific CD8^+^ T cells strongly ameliorated antitumor efficacy by improving IFNγ production and lowering inhibitory receptor expression, MAF overexpression reduced intratumoral accumulation and antitumor effector function. MAF is induced by TGF-β and IL-6, thereby integrating immunosuppressive cues from the TME to converge on suppression of antitumoral functions of TILs. Although TGF-β and IL-6 are not exclusively present in cancer settings, in some tumor types, such as melanoma, they are strongly upregulated and represent a major immunosuppressive axis exploited by cancer cells and tumor-associated immune cells to promote tumor persistence. MAF represents therefore a microenvironment-induced transcriptional regulator to promote exhaustion and to lock tumor-specific TILs in dysfunctional states (98).

Further evidence for the role of MAF in tumor-induced exhaustion comes from Chihara et al., where the authors identified c-MAF and PRDM1 (Blimp1) as cooperative regulators driving co-expression of inhibitory receptors in TILs (99). In this study, the authors analyzed the transcriptomic and proteomic profile of CD8^+^ and CD4^+^ TILs identifying and experimentally validating key transcriptional regulators of the inhibitory modules during tumor-induced exhaustion. They show that in this context, PRDM1 and MAF are upregulated by IL-27 and they converge in reinforcing terminal exhaustion programs by increasing multiple co-inhibitory receptor expression. Genetic ablation of these two transcription factors resulted in alleviated exhaustion and stronger effector-like phenotype in TILs, finally leading to improved anti-tumor capacity.

Together, these studies position the transcription factor MAF as an important node integrating microenvironmental cues from the TME and translating them into tumor-driven T cell dysfunction. Mechanistically, diverse pathways have been identified to positively regulate expression of MAF in TILs, providing interesting and potentially therapeutically targetable pathways to better harness immunotherapeutic strategies.

### 3.6 Epigenetic imprinting and fixation

A defining feature of CD8^+^ T cell exhaustion is its epigenetic stability. Paired studies by Pauken et al. and Sen et al. demonstrated that exhausted T cells harbor a distinct enhancer landscape that is not reversed by PD-1 blockade. Although checkpoint inhibition transiently restores effector function, the underlying chromatin accessibility profile remains largely intact, indicating that exhaustion is epigenetically imprinted rather than purely signaling-dependent. These exhaustion-specific enhancers are enriched for motifs associated with chronic stimulation programs, distinguishing them from effector or memory states (11, 12). In tumors, this fixation is further reinforced by TOX. It was shown that TOX actively enforces exhaustion-associated chromatin accessibility in tumor-infiltrating CD8^+^ T cells, stabilizing inhibitory gene expression and restricting effector reprogramming. Together, these findings establish exhaustion as a lineage-like state, epigenetically encoded and resistant to simple checkpoint reversal, particularly within the tumor microenvironment (17).

The recognition that exhaustion is epigenetically fixed has reshaped immunotherapeutic strategies. Rather than viewing PD-1 blockade as a complete reset, emerging work emphasizes the need to modulate chromatin architecture itself. Yousif et al. highlight how epigenetic regulators, including histone modifiers, DNA methylation machinery and chromatin remodelers, coordinate the transcriptional rigidity of exhausted CD8^+^ T cells. Therapeutic strategies targeting these regulators aim to loosen exhaustion-specific enhancer networks, thereby increasing plasticity and restoring responsiveness to immunotherapy (100). It was then further argued that effective immunotherapy may require “rewriting history”, that is, reprogramming differentiation trajectories before exhaustion becomes terminally fixed. Their work emphasizes that progenitor-like exhausted T cells retain partial epigenetic flexibility, whereas terminally exhausted subsets exhibit deeply entrenched chromatin states. This distinction is particularly relevant in cancer, where persistent antigen exposure and metabolic stress accelerate fixation (101). Wong et al. describe the interplay between epigenetic regulation and metabolic constraints in shaping T cell fate. In tumors, hypoxia, nutrient deprivation, and suppressive cytokines reinforce chromatin configurations that limit mitochondrial function and effector gene accessibility. Thus, epigenetic and metabolic dysfunction are mechanistically intertwined (102).

Collectively, these studies suggest that successful tumor immunotherapy will likely require combinatorial approaches: checkpoint blockade to relieve inhibitory signaling, alongside epigenetic modulation to destabilize exhaustion-specific chromatin landscapes. By targeting the architectural foundations of dysfunction, rather than only surface inhibitory receptors, it may become possible to reprogram tumor-infiltrating CD8^+^ T cells toward durable effector or memory states.

## 4 Conclusion

CD8^+^ T cell exhaustion represents a conserved differentiation program induced by persistent antigen exposure, originally defined in chronic infection models but now firmly established as a central barrier to effective antitumor immunity (1, 3, 5). As highlighted throughout this review, mechanisms of exhaustion in tumors consist in pre-existing adaptive program reshaped under this unique hostile microenvironmental conditions. Sustained TCR signaling remains the initiating force, engaging NFAT-dependent rewiring and TOX-mediated chromatin remodeling (13–17). However, in cancer, this canonical pathway is amplified and stabilized by tumor-specific extrinsic pressures including hypoxia (32–39), mechanical stress (43), metabolic competition and ionic imbalance (49, 50, 52–54, 58, 61, 62), and heterogeneous antigen density (70–72). These cues do not merely suppress effector function transiently, they converge to imprint a durable transcriptional and epigenetic state. Studies focus both on endogenous TILs and on CAR T cells but do not compare directly the two cell types. To which extent the nature of the TCR vs CAR-T can influence the program of exhaustion remains to be clearly defined. However, most mechanisms described so far are common to both (22, 103, 104).

At the transcriptional level, tumors reinforce exhaustion through coordinated activation of signal-sensitive transcription factor networks. Chronic NFAT activity, including tumor-specific engagement of NFAT5 in response to osmotic and ionic stress, integrates antigen persistence with microenvironmental constraints, deepening functional impairment (13, 73, 74, 76). The IRF family further exemplifies how tumors exploit signal interpretation pathways: sustained IRF4 activity calibrates differentiation according to chronic TCR strength (77–81), IRF8 shapes exhaustion-associated chromatin topology within both T cells and the tumor niche (82, 84, 93), and IRF2 translates prolonged interferon exposure into stable dysfunctional programs (87). These IRF-centered circuits illustrate how tumors repurpose inflammatory and differentiation machinery to consolidate exhaustion rather than memory or durable effector states. Chromatin remodeling emerges as the decisive layer of fixation. The BATF/IRF4 axis acts downstream of chronic signaling to establish exhaustion-compatible accessibility landscapes (12, 81, 92–95), while NR4A family members reinforce inhibitory transcriptional modules and impose metabolic restraint in tumor-infiltrating T cells (22, 96, 97). Crucially, exhaustion is epigenetically imprinted. Exhausted T cells harbor stable enhancer repertoires that are not fully reversed by PD-1 blockade (11, 12), and TOX enforces tumor-specific chromatin accessibility (17). Thus, checkpoint inhibition transiently reinvigorates function without fundamentally resetting lineage identity. This epigenetic stability explains both the partial efficacy of immunotherapies and the frequent relapse associated with terminally exhausted populations.

A unifying principle therefore emerges: tumor-induced exhaustion results from the convergence of conserved chronic-stimulation circuitry with tumor-specific environmental reinforcement. Persistent antigen exposure initiates the program, but hypoxia, metabolic stress, mechanical forces, interferon gradients, and chromatin remodeling networks stabilize and entrench it. The tumor microenvironment functions not only as a suppressive niche but as an active architect of T cell lineage fate. Therapeutically, this framework argues for multi-layered intervention strategies. Effective reprogramming of tumor-infiltrating CD8^+^ T cells will likely require simultaneous modulation of antigen signaling thresholds, metabolic resilience, stress-sensing pathways, and chromatin architecture. Approaches targeting BATF-, IRF-, NR4A-, or NFAT-centered circuits, combined with metabolic engineering and checkpoint blockade, may destabilize exhaustion at its structural core rather than merely relieving inhibitory receptor signaling. Importantly, preserving or expanding progenitor-like exhausted populations while preventing terminal epigenetic fixation may represent the most promising avenue for durable tumor control (20, 21, 25, 26, 105, 106). In conclusion, tumor-specific CD8^+^ T cell exhaustion is best understood as a layered, environmentally reinforced lineage program. By dissecting the intersection between conserved chronic stimulation pathways and tumor-imposed transcriptional and epigenetic constraints, we move closer to rational strategies capable of transforming exhaustion from a barrier into a therapeutically tractable state.
